# The Extremely Preterm Infant: Ethical Considerations in Life-and-Death Decision-Making

**DOI:** 10.3389/fped.2020.00055

**Published:** 2020-02-26

**Authors:** Susan Albersheim

**Affiliations:** Division of Neonatology, Department of Pediatrics, University of British Columbia, BC Women's Hospital, Vancouver, BC, Canada

**Keywords:** extremely preterm infant, neurodevelopmental impairment, decision-making, personhood, justice as fairness, parental discretion, harm principle, shared decision-making

## Abstract

Care of the preterm infant has improved tremendously over the last 60 years, with attendant improvement in outcomes. For the extremely preterm infant, <28 weeks' gestation, concerns related to survival as well as neurodevelopmental impairment, have influenced decision-making to a much larger extent than seen in older children. Possible reasons for conferring a different status on extremely preterm infants include: (1) the belief that the brain is a privileged organ, (2) the degree of medical uncertainty in terms of outcomes, (3) the fact that the family will deal with the psychological, emotional, physical, and financial consequences of treatment decisions, (4) that the extremely preterm looks more like a fetus than a term newborn, (5) the initial lack of relational identity, (6) the fact that extremely preterm infants are technology-dependent, and (7) the timing of decision-making around delivery. Treating extremely preterm infants differently does not hold up to scrutiny. They are owed the same respect as other pediatric patients, in terms of personhood, and we have the same duties to care for them. However, the degree of medical uncertainty and the fact that parents will deal with the consequences of decision-making, highlights the importance of providing a wide band of discretion in parental decision-making authority. Ethical principles considered in decision-making include best interest (historically the sine qua non of pediatric decision-making), a reasonable person standard, the “good enough” parent, and the harm principle, the latter two being more pragmatic. To operationalize these principles, potential models for decision-making are the Zone of Parental Discretion, the Not Unreasonable Standard, and a Shared Decision-Making model. In the final analysis shared decision-making with a wide zone of parental discretion, which is based on the harm principle, would provide fair and equitable decision-making for the extremely preterm infant. However, in the rare circumstance where parents do not wish to embark upon intensive care, against medical recommendations, it would be most helpful to develop local guidelines both for support of health care practitioners and to provide consistency of care for extremely preterm infants.

## Introduction

Care of the premature infant has been an area of great medical progress since the term neonatology was first introduced, in 1960. With improved technology we can care for smaller and less mature infants than ever imagined, but care of the Extremely Preterm Infant (EPI), <28 weeks' gestational age, has also engendered a great deal of moral distress for parents^1^ and for health care providers (HCPs), particularly related to complications of neurodevelopmental impairment (NDI). There have been several epochs during which concerns of NDI have directly influenced life-and-death decisions. Although infanticide has been practiced since the beginning of time, even 100 years ago lethal eugenics of defective newborns was publicly discussed and promoted through the eugenics movement (3), until the Nazi Children's Euthanasia Program came to light, where an estimated 5,000 German children with mental or physical handicaps were killed because of their disabilities, “a life unworthy of life.” In general, the backlash to the atrocities of the Holocaust resulted for a time in a quiescence of further conversations about eugenics and euthanasia.

Improvements in survival of premature infants, based on a better understanding of pathophysiology and the equipment to treat these babies, again raised concerns related to neurodevelopmental outcomes (4), which resulted in discussions of whether we ought to treat infants who are anticipated to have NDI.^2^ Previously we could not treat these premature infants, and could only pity them (5). In the early years of neonatology it was not uncommon if there were concerns of NDI that ventilators were discontinued, resulting in death of the baby. This continued until the aftermath of the Bloomington Baby Doe, a newborn with Down syndrome, tracheoesophageal fistula and esophageal atresia (Baby Doe, 1982), and Baby Jane Doe, a baby with meningomyelocele, hydrocephalus, and microcephaly (Baby Jane Doe, 1983), both of whom died after decisions were made not to treat due to concerns of quality of life. These cases went to the courts, eventually resulting in the Baby Doe Law in the United States (6). This was an amendment to the 1984 Child Abuse Law which extended the definition of child abuse to the withholding of fluids, nutrition, and medically indicated treatment from disabled children.

Thereafter, standard of practice in management discussions for critically ill neonates could not include NDI, even in Canada where the Baby Doe Law did not apply.^3^ Subsequently fully informed consent included discussion of neurodevelopmental outcomes. For management of the EPI this included defining the “threshold of viability”^4^ and providing standardized and up-to-date outcome statistics, based on gestational age. Over time, neonatologists, accused of gestational ageism (9), came to realize that other considerations such as birth weight, sex, singleton birth, antenatal corticosteroids (10), active resuscitation (8), chorioamnionitis, congenital malformations, and prolonged rupture of membranes influence outcome, and are relevant in decision-making for the EPI.

In neonatology today parents and HCPs generally agree about the management of the EPI, but occasionally parents request active resuscitation against the recommendations of HCPs. And although it is extremely rare, there are times when parents do not wish to embark upon intensive care, contrary to the recommendations of the HCPs. It is also controversial at which gestational age it would be standard of practice to resuscitate the EPI against parental wishes. When the aforementioned disagreements arise, both parents and HCPs may experience significant moral distress.

Realizing how the pendulum has swung over the short history of neonatology, with different attitudes to this age group, I will review ethical considerations in the management of the EPI, beginning with the status of the EPI, and then review models of decision-making, with particular attention to the situation where contrary to the recommendations of HCPs, parents elect not to start intensive care. I conclude that although the EPI ought to be treated similarly to older children, there are compelling reasons to accord greater deference to parents in decision-making.

## Status of the Extremely Preterm Infant

A critical question is whether the EPI is the same as any other pediatric patient, or whether the EPI should be attributed a different status and therefore can be treated differently, which has been how the EPI has historically been treated. If the EPI has the same status, in terms of personhood, then all treatment decisions should be in keeping with standards of practice for other pediatric age groups. Understood in terms of justice as fairness, that equals ought to be treated equitably, all children in similar circumstances should be treated similarly. Janvier et al. have argued that premature infants are devalued in our society, even compared to older patients who have a worse prognosis (11), and therefore attributing a lesser moral status to premature infants would be morally wrong (12).

Rieder, on the other hand, draws a “distinction between rescuing and continuing or taking over creation,” and as there are different reasons for rescuing and creating, “they do not exert the same normative force” (13). That is, resuscitating EPIs who would have otherwise not survived, may confer a different moral status on the EPI, which will be explored below.

The first consideration to address is whether the issue is the moral status of the EPI or the resuscitation *per se*? In terms of resuscitation, we treat patients who have survived resuscitation as we would patients who have not been resuscitated. Length of cardiac compressions and number of cardiac arrests may influence future decision-making, but surviving resuscitation, particularly in a situation where a patient would be anticipated to require resuscitation, such as a near-drowning, would generally result in full treatment subsequently. Therefore, the question is whether there are other reasons that an EPI may have a different status from older children.

First, a common belief is that the brain is a privileged organ, and as such having a severe NDI may be a compelling reason not to provide intensive care. An example would be an extremely ill EPI with perforated necrotizing enterocolitis, who also has extensive bilateral grade 4 intraventricular hemorrhages and post-hemorrhagic hydrocephalus. In this scenario the HCP may say that the situation is futile. It is essential to differentiate whether this is physiological futility, where a specific treatment will not be effective in treating the condition, such as surgery to remove the necrotic intestine and subsequent surgery for hydrocephalus, or whether this is an evaluative futility, due to concerns of long term severe NDI. The HCP may believe that a shunt to treat the hydrocephalus is not warranted because the quality of life would be so poor that the baby would be better off not “suffering”^5^ in the Neonatal Intensive Care Unit (NICU), and beyond the NICU. Interestingly, interviewing HCPs, adolescents (who had been either premature infants born extremely low birth weight (<1,000 g) or full term infants), as well as their parents, about hypothetical health states, Saigal et al. identified that both groups of parents as well as adolescents value health-related quality of life with severe disability higher than HCPs (14). Moreover, although the potential for NDI is not sufficient to warrant redirection of care in older pediatric patients, severe neurological injury, with anticipated short and long term significant NDI, is a relevant consideration. Therefore, as with older children, severe neurological injury, similarly to significant damage to any other vital organ, not merely the potential for organ damage, is an important consideration in life-and-death decision-making. However, having a NDI does not confer a lesser status on a person.

Secondly, a major feature of the management of the EPI is that there is a great deal of medical uncertainty in terms of outcomes. It is impossible to prognosticate with any degree of certainty for the individual patient, be that prior to birth, at the time of a live birth, or for a sick neonate in the NICU (10, 15). There are many patients encountered in medicine who present with a condition for which there is a great deal of medical uncertainty. When there is a high chance of death or severe NDI intensive care is not ethically obligatory. However, in older children in the face of medical uncertainty, intensive care would be initiated pending clear evidence of prognosis in terms of short-term and long-term benefits and harms of the proposed treatment (16). For the EPI, as with older pediatric patients, initiating resuscitation and intensive care, pending more information about response to treatment and neuroimaging, at which time a more informed choice for available treatment options could be made, is reasonable. Medical uncertainty is not unique to neonatology, but does provide a rationale for parental decision-making authority.

Thirdly, families deal with the consequences of treatment decisions. Parents are also best situated to appreciate the religious and cultural community in which their child will live, and hopefully flourish. However, there may not be adequate resources to deal with all the post-NICU needs of the EPI with significant complications of prematurity, so parents are left to deal with the psychological, emotional, physical, and financial burden of caring for their children.^6^ A child with significant NDI requires more resources, and on a rare occasion parents will place their child into foster care or give their child up for adoption, due to an inability to provide the level of care required. One may posit that the harms of foster placement, possibly with multiple care providers, may outweigh the benefits to the child, but there is a dearth of literature on outcomes of EPIs with or without NDI in foster care settings, and therefore we are unable to predict the harms or benefits in terms of quality of life, particularly compared to the harms or benefits of being dead, which are unknowable. This does not identify EPIs as different from older pediatric patients, but it highlights the importance of parents in decision-making and provides justification for a fairly wide zone of parental discretion in decision-making (18).

Fourth is what the EPI actually looks like. The EPI has been called a “fetusoid,” a pejorative term, which demonstrates that some HCPs have viewed the EPI as being in a liminal phase, between a fetus and a fully developed human being. In our society abortion is legal, and in the not too distant past there was often nothing that could be done to treat extreme prematurity. Thus, it is not surprising that it is easier emotionally to discontinue intensive care on an EPI soon after birth, a baby who looks more like a fetus, very fragile and being kept alive by technology, that is much larger than the baby, than later in the NICU course. Rieder claims that if an EPI is born at 55–60% of a full gestation resuscitation is not rescuing a child by “preventing a fatal drop in well-being…from a normal baseline,” but rather “artificially continuing gestation…taking over the creative process that gets a child to a normal baseline” (13). However, there is a clear separation between the fetus and the newborn, based on physiological differences; the “bright line of birth” (19). Rieder states that when rescuing a patient the potential benefits are weighed against the potential burdens of the intervention, whereas in the creative process the potential burdens (such as becoming pregnant when infected with a virus that may cause severe damage to the fetus) are more compelling than potential benefits of procreation (that one would be an ideal parent); hence an “asymmetry” exists (13). Rieder supports the concept of a creative process with examples of greater attachment to a child than to an embryo, also highlighting an emerging personhood (13). A late preterm loss is more distressing than an early loss, as a loss of someone whom we have known for a long time is more distressing than the death of an acquaintance, but that does not mean that they are of lesser status. Although Rieder presents an intriguing argument for a different status for the EPI, it is not supported physiologically.

Therefore, despite appearances it is clear that the EPI is a human being and the HCP has a duty to care for the EPI, be that immediately after birth or closer to term corrected age, when the ex-EPI looks more like our vision of a newborn baby.^7^

Fifth, our commitment to the EPI develops over time, and as the EPI and our relationship with the EPI grows and develops, she/he truly becomes a member of a family, and the liminality which we may have conferred on the EPI in the NICU, is impacted by a developing relational identity. This concept of a liminal phase in the NICU with a developing relational identity is an understandable but artificial construct which is not a valid reason to confer a different status on the EPI.

Sixth, a common belief has been that the only good outcome would be a life independent of technology. And although a life of intensive care may not be considered a reasonable quality of life for some people, there are many pediatric and adult patients who spend a very long time in hospital, technology-dependent, or today may go home with ventilators, feeding tubes, dialysis, etc. The technology itself is not a reason to consider that a patient has a different status than a non-technology-dependent patient.

Last is the issue of timing, as in Canada treatment decisions, including mode of delivery, can only be made by the pregnant woman prior to birth, and emergency application to the court at the time of birth is generally not a feasible option. However, after birth if informed capable parents decline resuscitation for their EPI, which in the opinion of the HCP is beneficial and therefore warranted for the baby, it is standard of care to provide life-saving treatment in an emergency situation, with an application to Child Protective Services for continued intensive care. Although rare, it is an important issue which must be openly discussed with parents, for transparency and for the sake of the therapeutic relationship. In the final analysis, it would appear that there is no clear or compelling reason to assign a different status to EPIs, and therefore they ought to be treated like other pediatric patients, warrant the same respect (20), and are owed the same duties as older children. However, the degree of medical uncertainty inherent in decision-making, the fact that parents know the cultural and religious home environment, and will be caring for their child, who may have special needs, for a very long time, places parents in a privileged position. I now turn to decision-making for the EPI, and begin with ethical principles that may guide these decisions.

## Ethical Principles

Historically decision-making for children has been based on the Best Interest Principle. However, best interest is very difficult to define. Hester considers best interest in a practical sense as a “reasonable person” standard, and identifies various interests of the newborn: medical, pain and suffering, and future potential interests (21); at its' most basic the right to life, without significant pain and suffering, and meaningful relationships with others (22). Despite attempts by various bioethicists to more clearly articulate and to operationalize best interest, it is still extremely difficult to adjudicate for the EPI. Therefore, the decision-maker is integrally linked with the best decision, and parents have a moral claim to be decision-makers for their children. Professional organizations, such as the Canadian Pediatric Society confirm that capable, informed parents are considered the best surrogate decision-makers for their not-yet-competent children (23).

However, Ross identifies that we in fact do not expect a parent to be the “best” parent, but rather a “good enough” parent^8^ (20). Parents are the appropriate decision makers for their child's interests, which includes standard medical care, but also incorporate the social, psychological and emotional interests of the child. Thus, decision-making is far more nuanced than merely evaluating a treatment decision for an EPI. This includes other factors particular to the individual EPI and their intimate family. Ross proposes a model of constrained parental autonomy, based on respect for the child, with state intervention for the following health care decisions: (a) if parental refusal places the child at high risk of “serious and significant” mortality or morbidity, and “treatment is of proven efficacy with high likelihood of success”; (b) if parents authorize treatment that is “virtually futile and inhumane” (20).

Similarly, Diekema identified the Harm Principle as the threshold for state intervention (25). This is particularly relevant in considering two aspects of care for the EPI. The first relates to the potential harm associated with NICU treatment, be that procedural pain or significant discomfort.^9^ Diekema identifies that “…we should be reluctant to override parental wishes when therapy itself poses grave risks…” (25) Painful procedures, particularly multiple painful procedures, at random intervals and often without someone to comfort the baby, not only results in a very poor quality of life in the short term,^10^ but has been shown to have a longterm impact on brain development (27). The second issue addressed by the Harm Principle relates to situations where parents disagree with the recommendations of the HCPs to embark upon intensive care. Rather than trying to identify what is best or optimal for a child, the Harm Principle focuses on whether the child is at significant risk of serious harm, and the potential benefits of the intervention significantly outweigh the potential burdens of the option chosen by the parents. However, in the clinical setting there will be an element of interpretation by physicians, as to the chance of an outcome with or without the proposed treatment, and frequently percentages given are guesstimates at best, rather than current local statistics for a specific child. In addition physicians have all the same human frailties of other human beings, so that the neonatologist may be very optimistic in counseling when coming on service well-rested on a Monday morning, but after a week caring for very sick EPIs, some of whom have had serious complications or may have died, that same neonatologist may be far less optimistic by the end of the day Friday. Therefore, relying on “expert opinion” rather than on decision-making models or objective guidelines is problematic, and although the Harm Principle is more practical than Best Interest Standard, it is still difficult to operationalize for the EPI, but does highlight the importance of appreciating the potential harms, not just benefits of intensive care in counseling parents, particularly when considering overriding parental decision-making authority.

## Models for Decision-Making

Based in the Harm Principle, Gillam (2016) proposes the Zone of Parental Discretion (ZPD) for ethical deliberation when parents disagree with the recommendations of the health care team (18). And although decision making is based on parental values, Krick suggests that the ZPD is affected by the degree of prognostic uncertainty; the more uncertain, the wider the ZPD (28). However, we are still faced with the challenge of identifying probable significant harm.

A similar framework for surrogate decision-making, developed by Rhodes and Holzman, is called the Not Unreasonable Standard, which consists of three boxes (29). In the first box it would be Unreasonable to embark upon intensive care if there is no chance that we will be effective in treating the condition. For example, implementing intensive care at 20 weeks' gestational age provides greater burden than benefit, and is experimental, since equipment is not available to care for a baby that small, and there have been no documented survivors. However, when there is a chance of survival, informed, capable parents can choose intensive care treatment.

In the middle box, it would Not be Unreasonable to either provide intensive care or not provide intensive care. Parents, as the best surrogate decision-makers for their child, have different world views, different religious beliefs, and different personal and economic capacities. Society may provide some funding for the long term care of the EPI, but parents will primarily deal with the consequences of these decisions, and therefore it is incumbent upon HCPs to provide a wide ZPD in the face of significant medical uncertainty (18).

The third box incorporates situations where it would be Unreasonable not to provide treatment, which “promises a likely and significant medical benefit, whereas refusal of treatment is very likely to result in significant harm” (29). Again this is adjudicated by the expert, the physician, and is not an objective standard. The question is what chance of survival or chance of survival without severe NDI^11^ would entail “significant medical benefit”? Risks and benefits are different for each individual, as is interpretation of statistics (31).

Appreciating the complexity of decision-making in the NICU and the unique nature of each family, Lantos recommends the Shared Decision-Making model (32). In Shared Decision-Making, HCPs “share the best available evidence,” discuss “options,” (33) and “help parents discern their own values and ethical commitments as they face an unanticipated situation and a series of life-altering decisions” (34). This model holds much appeal conceptually, but unless employed as recommended (35, 36), when there are intractable differences between parents and HCPs, may default to decision-making by medical authority, a paternalistic approach which belies the shared nature of such a decision. Lantos states that when treatment is clearly beneficial, according to medical experts, a baby's right to treatment outweighs parental decision-making authority, and when the benefits of treatment are less clear, parents should decide for their not-yet-competent children (32). Clearly beneficial may be difficult to ascertain in implementing a threshold for parental decision-making authority. For an older child when parents disagree with the HCPs, pediatricians rarely refer to Child Protective Services. Since we are saying that the EPI ought to be treated like older children is there a standard of care for older children, which would justify overriding parental choice? In children with cancer for example, an assessment is made of the chance of survival, the burden or toxicity of the treatment and the anticipated long term outcomes. The more burdensome the treatment the better the long term outcomes will need to be to justify overriding parental decision-making authority. For the EPI intensive care is definitely burdensome, so the question is the chance of survival free of severe long term impairment which would warrant intensive care treatment as standard of care, and if treatment was not provided would constitute medical neglect?

It may appear that we have come to an impasse considering the aforementioned ethical principles and models of decision-making. However, it may be possible to move forward if we can agree about standards for implementing intensive care even against parental wishes. Although neonatologists appreciate that there are important considerations beyond gestational age (9). they continue to request standardized periviable age-based guidelines (37, 38). A major impetus for guidelines is to provide a standard for what would be considered significant medical benefit, as well as providing equitable care for all EPIs within a geographical area, with consistency in counseling from Neonatologists and between Neonatologists and Perinatologists (39). Kaempf et al. acknowledge the importance of guidelines in decision-making for EPIs and that Shared Decision-Making is particularly useful (40). However, Kaempf and Dirksen identify that on rare occasions decisions, which are more appropriate for an individual family situation, may be contrary to the guidelines (40, 41). Therefore, it would appear that the process of Shared Decision-Making with a wide ZPD, may best support parents, promoting a trusting relationship between parents and HCPs, and that local guidelines, which must be understood as guidelines not as policy, would support HCPs in this process.

## Conclusion

Neonatology has come a long way in a relatively short period of time. Upon careful consideration, it is clear that the EPI ought to have the same status as older pediatric patients and is due the same respect. In general parents are the best surrogate decision-makers; as an intimate family they care about their children, support and deal with the outcomes of extreme prematurity, and should be accorded a wide zone of parental discretion. The Harm Principle is a reasonable standard for decision-making when parents disagree with the recommendations of HCPs, particularly when parents do not wish to embark upon intensive care. A truly Shared Decision-Making model would inform parents fully, acknowledging the unique world view of parents, and help them come to a decision. The threshold for embarking on intensive care should be guided by physiological futility. The limit to parental decision-making authority ought to be where the outcome is clearly beneficial, with a low likelihood of severe NDI. It is important to define these limits, developing local guidelines to provide standards of practice, which will support HCPs in counseling parents. At times guidelines may result in embarking upon intensive care with daily review of the direction of care. Transparency and regular communication with parents will invariably strengthen the therapeutic relationship, minimizing conflict, which historically has resulted in applying to the court system, considered the least desirable option, given the importance of a trusting relationship for the care of the EPI in the NICU and after discharge.

## Author Contributions

The author confirms being the sole contributor of this work and has approved it for publication.

### Conflict of Interest

The author declares that the research was conducted in the absence of any commercial or financial relationships that could be construed as a potential conflict of interest.

## Footnotes

^1^Foe et al. have suggested that it may be more common for parents to experience Moral Schism: “…a genuine uncertainty regarding a value-based decision that is accompanied by emotional distress.” (1) Moral distress has commonly been appreciated as the discomfiture associated with knowing the right thing to do but not being able to follow this course of action (2).

^2^Although the language of treating versus not treating neonates was previously commonplace, today we refer to treatment with intensive care or treatment with palliative or comfort care.

^3^In fact, the Baby Doe rules only referred to federal funding requirements for states to receive grants (7).

^4^“Threshold of Viability” is a potentially biased term, which may result in a self-fulfilling prophesy (8), and therefore throughout this paper I have rather discussed the management of the EPI (Extremely Preterm Infant).

^5^The word “suffering” is used in reference to the neonate, in clinical practice and in the literature. Suffering refers to physical pain, as well as mental or emotional anguish, but it is an emotive word. One would not say that a patient is “suffering” from post-operative pain, but rather call it pain and treat it. Likewise, in the neonate who may well feel pain, which should be treated appropriately, the baby is probably not suffering, although the parents/family and HCPs may experience suffering. On the other hand a baby in the NICU may experience more distress than an older child because it is not possible for a baby to understand what is happening.

^6^In this paper, decision-making for the EPI refers to management in western high-resource NICUs. However, decision-making in low-resource settings may also be morally-challenging, particularly in countries such as India, where the “well to do” and the “increasingly affordable middle class” can access the most advanced neonatal care, while the poor may not receive basic neonatal care (17). Due to the scope of this paper I have not addressed the cost of intensive care, but it is an important consideration, particularly in low-resource settings, as highlighted by Nagesh and Razak (17).

^7^Janvier et al. identified that some authors (P Singer, HT Engelhardt) judge that neonates do not obtain full personhood until 2 months (12). There is no physiological support to this arbitrary distinction. However, it speaks to the belief in the concept of a liminal phase, but does not justify it.

^8^Blustein refers to this concept as “satisficing parentalism” as opposed to “optimizing parentalism”: “that something less than the best for children can still be good enough” (24).

^9^A baby born less than 29 weeks' gestation may experience 300 or more painful procedures over their NICU stay (26).

^10^Previously it was believed that due to the immaturity of the nervous system that premature babies did not feel pain, and therefore inadequate or no anesthetic was given for surgery. We now know that babies <20 weeks' gestation can feel pain, but we are just beginning to understand the ramifications on the developing nervous system of what the NICU experience entails (27).

^11^Studies of parental input have identified that severe NDI is a relevant consideration (30).

## Abbreviations

EPI: Extremely Preterm Infant
HCP: Health Care Provider
NDI: neurodevelopmental impairment
NICU: Neonatal Intensive Care Unit
ZPD: Zone of Parental Discretion.
